# Association between Phthalate Metabolites and Risk of Endometriosis: A Meta-Analysis

**DOI:** 10.3390/ijerph16193678

**Published:** 2019-09-30

**Authors:** Wei Cai, Jule Yang, Yini Liu, Yongyi Bi, Hong Wang

**Affiliations:** School of Health Sciences, Wuhan University, Wuhan 430071, China; 18844504051@163.com (W.C.); yjl13525985915@163.com (J.Y.); 18655911293@163.com (Y.L.); yongyib@aliyun.com (Y.B.)

**Keywords:** phthalates, endometriosis, meta-analysis

## Abstract

*Objective*: The association between phthalates and endometriosis risk is inconclusive. This meta-analysis aims to evaluate the association between five different phthalate metabolites and endometriosis, based on current evidence. *Methods:* The literature included PubMed, WOS (web of science), and EMBASE, published until 3 March 2019. We selected the related literature and evaluated the relationship between phthalates exposure and endometriosis risk. All statistical analyses were conducted with STATA version 12.0. *Results*: Data from eight studies were used in this review. The results of this analysis showed that mono-(2-ethyl-5-hydroxyhexyl) phthalate (MEHHP) exposure was potentially associated with endometriosis (OR = 1.246, 95% CI = 1.003–1.549). We have not found positive results in mono(2-ethylhexyl) phthalate (MEHP), monoethyl phthalate (MEP), monobenzyl phthalate (MBzP) and mono(2-ethyl-5-oxohexyl) phthalate (MEOHP) analyses (MEHP: OR = 1.089, 95% CI = 0.858–1.383; MEP: OR = 1.073, 95% CI = 0.899–1.282; MBzP: OR = 0.976, 95% CI = 0.810–1.176; MEOHP: OR = 1.282, 95% CI = 0.874–1.881). In subgroup analyses for regions, the associations were significant between MEHHP and endometriosis in Asia (OR = 1.786, 95% CI = 1.005–3.172, I² = 0%), but not in USA (OR = 1.170, 95% CI = 0.949–1.442, I² = 45.6%). *Conclusions*: Our findings suggested a potential statistical association between MEHHP exposure and endometriosis, particularly, the exposure of MEHHP might be a potential risk for women with endometriosis in Asia. However, positive associations between the other four Phthalate acid esters (PAEs) and endometriosis was not found. Given the weak strength of the results, well-designed cohort studies, with large sample sizes, should be performed in future.

## 1. Introduction

Phthalate acid esters (PAEs) are mainly composed of dialkyl esters, or alkyl and aryl esters of orthophthalic acid (1,2-dicarboxylic acid) [1,2]. They affect human health to some extent, while some of them disrupt endocrine function and alter hormone activity in animals [3,4]. In recent years, the source of PAE uptake in humans, which includes personal care products, cosmetics, toys, home furnishings, nutritional supplements to pharmaceuticals, insecticides and medical instruments, have also been reported [5,6,7]. Studies have shown that some phthalate metabolites have been detected in nearly 80 percent of the population in the United States, indicating widespread PAEs exposure [8].

Over the past decade, a number of studies have been conducted to explore the relationship between reproductive problems in humans and PAEs [9,10]. Studies have shown that women are more likely to be exposed to PAEs through products such as perfume, cosmetics, and personal care products [11,12]. Because endometriosis is a common chronic gynaecological disorder associated with pelvic pain and infertility [13], it was important to explore the relationship between PAEs and endometriosis. Although, recent studies have shown that PAEs may have a certain influence on the development of endometriosis [14,15,16,17,18,19], there are also studies that disagree or suggest a lack of consistency in their results [20,21]. In addition, there are a wide variety of PAE metabolites, and their relationship with endometriosis is inconsistent [20,22,23]. PAEs, such as dimethyl-phthalate (DMP), di(2-ethylhexyl)-phthalate (DEHP), and dibutyl-phthalate (DBP), are commonly found in the blood add tissues of workers in industry, while phthalate metabolites, such as mono- (2-ethyl-5-hydroxyhexyl) phthalate (MEHHP), mono(2-ethylhexyl) phthalate (MEHP), monoethyl phthalate (MEP), monobenzyl phthalate (MBzP), and monobutyl phthalate (MBP), are often found in the blood and tissues of the general population. For example, Chao et al. [24] conducted a meta-analysis to analyze the relationship between the exposure to phthalate metabolites and male sperm quality. Lovekamp et al. [25] explored the effects of these phthalate metabolites on changes in the endogenous hormonal milieu.

Therefore, the purpose of the present study is to assess the potential effects of five phthalate metabolites exposure on endometriosis by a quality appraisal and a meta-analysis, based on human epidemiological studies.

## 2. Materials and Methods

### 2.1. Search Strategy

Searches were carried out using PubMed, EMBASE, and WOS (web of science) from 1 January 1995 to 3 March 2019. The literature search was conducted by two independent authors. We searched the keywords of phthalates (“phthalate”, “phthalic acid”, “phthalate ester” or “endocrine disruptors” or “diethyl phthalate” or “dimethyl phthalate ” or “dibutyl phthalate” or “di(2-ethylhexyl) phthalate” or “diisodecyl phthalate” or “diisononyl phthalate ” or “benzyl butyl phthalate” ) AND endometriosis (“endometriosis” or “endometrioses” or “endometrioma” or “endometriomas”).

### 2.2. Study Selection Condition

We included studies according to the following criteria: (1) Papers investigated phthalates and their metabolites; (2) one outcome was endometriosis; (3) cohort, case-control or cross-sectional studies; (4) odds ratio (OR) or relative risk (RR) were offered for the associations between phthalate exposure and endometriosis; (5) published in English.

Two authors (Cai and Yang) independently examined all studies. Disagreements were resolved through discussions.

### 2.3. Data Extraction

The following data were collected: Authors, country, year; number of cases; study design; types of sample; exposure assessed, OR with 95% CIs. The data are available to all readers as required.

### 2.4. Assessment of Study Quality

We evaluates the studies in our meta-analysis based on the Newcastle–Ottawa Scale (NOS) [26,27]. Assessment items included case selection, comparability of cases, assessment of outcome, and ascertainment of exposure. The maximum score was 9 in cohort or case-control studies and 10 in cross-sectional studies. For case-control or cohort studies, the total scores are divided into three grades: low (0–3), medium (4–6) and high (7–9), and for cross-sectional studies, the total scores are divided into three grades: low (0–3), medium (4–7) and high (8–10). Quality assessment was extracted by two authors (Cai and Yang), and disagreements were resolved through consensus.

### 2.5. Statistical Analysis

Data in this meta-analysis were analyzed using STATA, version 12.0 (Stata Corp LP, College Station, TX, USA), OR with 95% corresponding CI were used to assess the relationship between phthalate exposure and endometriosis. To estimate the heterogeneity among studies, we used chi-squared test and Cochran Q score (reported as I^2^) with corresponding *p*-values. We used the random-effect model in our meta-analysis and conducted a subgroup analysis with the study population (laparoscopic/laparotomy population or general population), the study design (case-control, cohort or cross-sectional study), and the study region (USA or Asia).

Additionally, a sensitivity analysis was conducted by excluding each study to evaluate the influence of each individual study on our pooled estimate. All this analysis was performed by STATA version 12.0 (Stata Corp LP, College Station, TX, USA). The publication bias was not evaluated because the number of included studies was not more than ten [28].

## 3. Results

### 3.1. Literature Search

**Figure 1:**
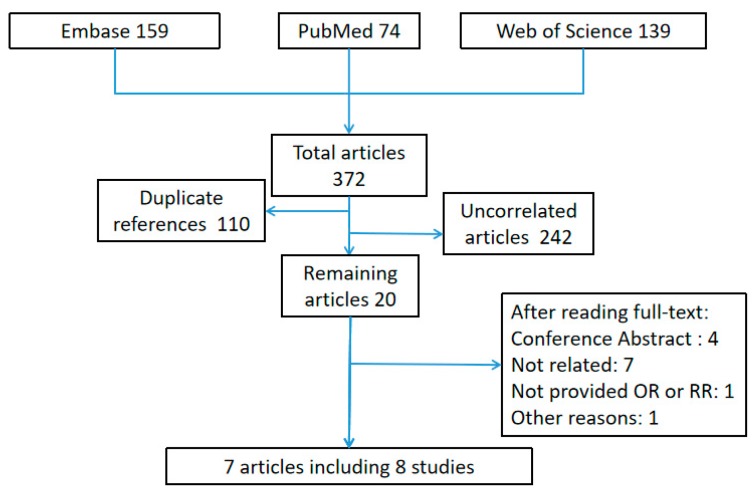
Literature search result (OR, odds ratio; RR, relative risk.).

Figure 1 shows the results of the literature search process, 372 studies were abstract from EMBASE, WOS (Web of Science) and PubMed. By screening of titles or abstracts, 242 studies uncorrelated to our study thus it had been excluded, 110 of all studies were duplicate references. After reading full-text, 7 articles [20,23,29,30,31,32,33], including 8 studies were included in our meta-analysis.

### 3.2. Study Characteristics

Table 1 shows the main characteristics of the 8 studies in our meta-analysis. Among these 8 studies, 4 studies were conducted in the USA, 2 studies were conducted in Korea, while the other 2 studies were conducted in China, Japan, respectively. In addition, the sample size in these studies ranged from 57 to 1107. Seven studies were about urinary test and the other study was about blood test. While, 5 studies were case-control designs, 2 studies were cohort designs and one study was cross-sectional design.

Based on the NOS, 5 of 8 studies scored 7, and the other 3 studies scored 6. Table 2 provided details of the quality assessment.

### 3.3. Meta-Analysis

**Figure 2:**
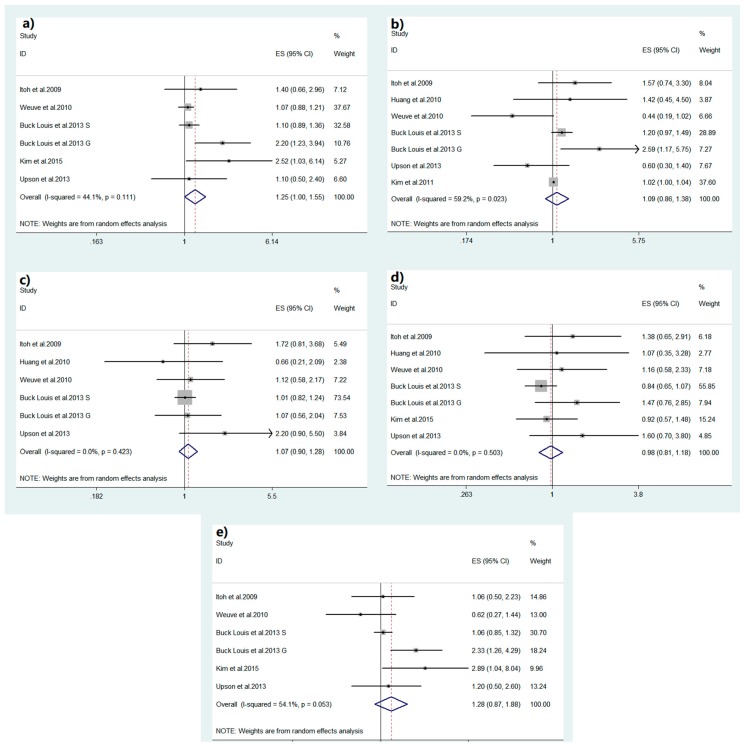
Forest plots of studies included in the meta-analyses: (**a**) MEHHP and endometriosis; (**b**) MEHP and endometriosis; (**c**) MEP and endometriosis; (**d**) MBzP and endometriosis; (**e**) MEOHP and endometriosis.

Pooled results suggested that MEHHP was significantly associated with the risk of endometriosis (OR = 1.246, 95% CI = 1.003–1.549, Figure 2a). No significant results were observed in MEP, MBzP, MEHP and MEOHP analyses (MEHP: OR = 1.089, 95% CI = 0.858–1.383, Figure 2b; MEP: OR = 1.073, 95% CI = 0.899–1.282, Figure 2c; MBzP: OR = 0.976, 95% CI = 0.810–1.176, Figure 2d; MEOHP: OR = 1.282, 95% CI = 0.874–1.881, Figure 2e). Random-effect models were adopted in addressing the association between MEHHP, MEHP, MEP, MBzP, MEOHP, and endometriosis.

### 3.4. Subgroup-Analysis

A subgroup analysis was conducted to assess the influences of study design, study population, region and sample on the estimation of overall OR.

#### 3.4.1. MEHHP Exposure and Endometriosis Risk

In subgroup analyses for regions, MEHHP was significantly associated with endometriosis in Asia (OR = 1.786, 95% CI = 1.005–3.172, I² = 0%), but not in USA (OR = 1.170, 95% CI = 0.949–1.442, I² = 45.6%). No specific relationships were identified between MEHHP exposure and endometriosis risk in the subgroups of the study design or population. The detailed results are summarized in Table 3.

#### 3.4.2. MEHP Exposure and Endometriosis Risk

In subgroup analyses for regions, MEHP was associated with endometriosis risk in Asia (OR = 1.020, 95% CI = 1.003–1.038, I² = 0%). However, the result was weak and additional future studies need to be included in the analysis. No specific relationships were identified between MEHP exposure and endometriosis risk in other subgroups. The detailed results are summarized in Table 4.

#### 3.4.3. MEP, MBzP, MEOHP Exposure, and Endometriosis Risk

Subgroup analyses for MEP, MBzP, MEOHP exposure were performed in Table 5, Table 6 and Table 7. No specific relationships were identified between these three metabolites and endometriosis risk in the subgroups.

### 3.5. Sensitivity Analysis

**Figure 3:**
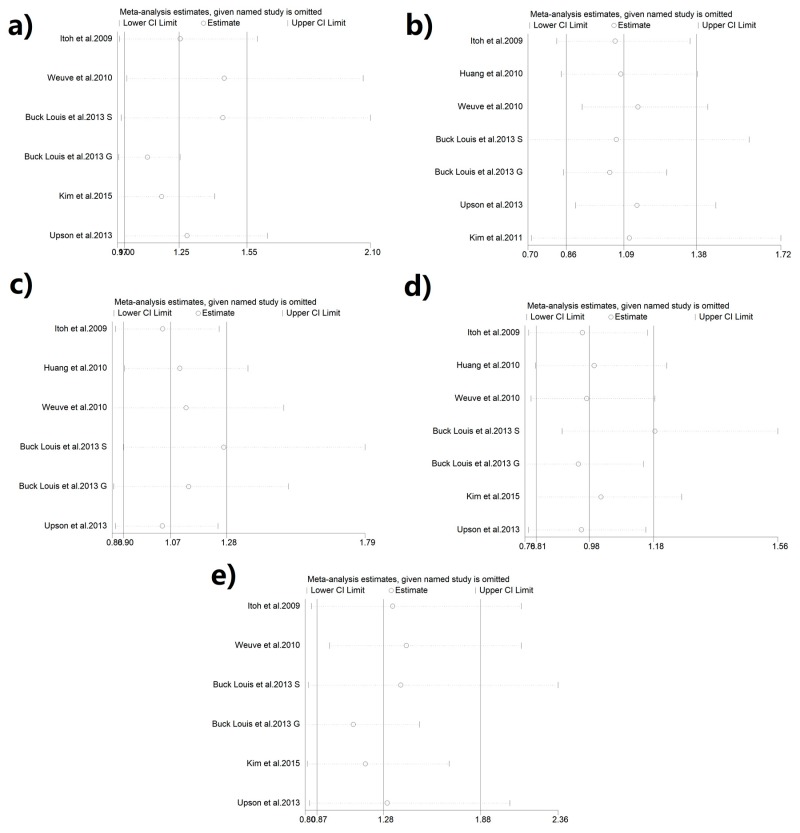
Sensitivity analysis plots: (**a**) MEHHP and endometriosis; (**b**) MEHP and endometriosis; (**c**) MEP and endometriosis; (**d**) MBzP and endometriosis; (**e**) MEOHP and endometriosis.

We conducted sensitivity analyses in order to exclude the influence of single study on the overall results. As Figure 3a–e shown, no single study significantly altered the overall OR.

## 4. Discussion

Phthalates have been shown to increase the rate of premature menopause in animals. Phthalates are estrogen and anti-androgen endocrine disruptors [34,35,36,37]. But the effects of phthalates on humans, especially reproductive development, are unproven [30,38]. However, whether the exposure to phthalates affects estrogen-related diseases, such as endometriosis, has not been addressed [14,15,16]. So our meta-analysis, which is a quantitative assessment of published data on the role of phthalates in endometriosis, was conducted to find a clear relationship between five different phthalates and endometriosis.

As far as we are aware, the current study is the first meta-analysis on the association of exposure to phthalates and endometriosis. In our meta-analysis, we investigated five types of phthalate metabolites, including MEHHP, MBzP, MEP, MEHP, and MEOHP. The findings of this meta-analysis indicated that only MEHHP was potentially associated with endometriosis. This result was consistent with a previous study that assessed the relationship between phthalates and endometriosis [16,32]. Besides, we did not observe the obvious relationships between the other four metabolites (MEHP, MEP, MBzP and MEOHP) and endometriosis. But several studies did not support this conclusion. For example, Cobellis et al. thinks phthalates may affect the development of endometriosis, and their results are the first to suggest a link between MEHP plasma levels and endometriosis [16]. Kim conducted a prospective case-control study in Korea and noted MEHHP and MEOHP played important roles in the risk of endometriosis [32]. However, several studies presented conflicting results [29,39]. Therefore, these results need to be further verified.

Subgroup analysis found that MEHHP exposure in Asia was associated with endometriosis. For the other metabolites, the results of subgroup analysis were not significant. For MEHHP group, after subgroup analysis was conducted by region, we found positive results in Asia but not in USA, suggesting that different countries have different levels of PAEs exposure, which plays a significant role, and different races also affect the outcome. Heterogeneity, that comes from regional diversity, race diversity, weight difference, sample size difference, age variation, health status, the concentration and duration of exposure of the study subjects or different detection methods, is relatively high for some meta-analysis and might have a potential effect when interpreting the synthesized results. After subgroup analysis was conducted by region, heterogeneity in the Asian group decreased, suggesting that regional factors may be related to heterogeneity, and that differences in PAEs exposure levels, in different countries, may be more important factors in this case.

Our meta-analysis has some advantages. Firstly, there has not been any meta-analysis on the relationship between phthalate metabolites and endometriosis risk before. Second, our meta-analysis identified the potential risk associated with five different phthalate metabolites and endometriosis. Third, this meta-analysis study was the ability to do a complete analysis on subgroups, such as types of studies (case-control, cohort, cross-sectional), region, subgroup of laparoscopic/laparotomy population, and general population, as well as the samples used (urine and plasma).

However, our meta-analysis also has some limitations: First, since there were only eight studies, the sample size is relatively limited. Second, there are potential confounding factors, such as age variation, weight difference, health status, BMI, and sample size differences. Although, some research data is based on the adjusted model, our results could be biased by the inherent limitations in the original studies. Third, different PAEs may coexist in some cases. And it has been found that MBzP, MEP, MEHP, and MBP may have some correlation, which indicates that they have a possible common sources of exposure [8]. Although, MEHHP was associated with endometriosis risk in our study, we could not rule out other phthalates that might influence the outcome.

## 5. Conclusions

Our findings suggested a potential statistical association between MEHHP exposure and endometriosis, particularly, the exposure of MEHHP might be a potential risk for women with endometriosis in Asia. No associations were identified between endometriosis with MEHP, MEP, MBzP, and MEOHP. Since the quality of the studies was moderate, it would be not appropriate to claim that these metabolites have no role in the progression of endometriosis. Our meta-analysis included only eight studies, involving fouor countries, and five common phthalate metabolites. Therefore, the available evidence is very limited, which may cause some bias in some aspects. Future studies should be conducted with larger sample size and higher number of phthalate metabolites.

## Figures and Tables

**Table 1:** Characteristics of studies included in the meta-analysis.

Study, Year	Country	Study Design	Age Range	Study Population	No. of Case/Control	Samples	Point Estimate	Categories of PAEs and Metabolites	Diagnostic Methods
Itoh et al. 2009	Japan	case-control	20–45	laparoscopic population	57/80	Urine	OR	MEP, MnBP, MBzP, MEHP, MEHHP, MEOHP	diagnosed using laparoscopy
Huang et al. 2010	China	case-control	27–45	laparotomy population	28/29	Urine	OR	MMP, MEP, MnBP, MBzP, 5oxo-MEHP, 5OH-MEHP, MEHP	based on the pathologic results of the presence of endometrial tissue outside the uterine cavity and within the myometrium with smooth muscle hyperplasia
Weuve et al. 2010	USA	cross-sectional	20–54	general population	87/1020	Urine	OR	MBP, MEP, MEHP, MBzP, MEHHP, MEOHP	according to the guidelines of the American Society for Reproductive Medicine
Buck Louis et al. 2013 S	USA	cohort	18–44	laparoscopic/laparotomy population	190/283	Urine	OR	MEP, MMP, MBP, MIBP, MECPP, MEHHP, MEOHP, MBzP, MEHP, MOP, MNP	Surgically visualized or pelvic magnetic resonance imaging (MRI)
Buck Louis et al. 2013 G	USA	cohort	18–44	general population	14/113	Urine	OR	MEP, MMP, MBP, MIBP, MECPP, MEHHP, MEOHP, MBzP, MEHP, MOP, MNP	Surgically visualized or pelvic magnetic resonance imaging (MRI)
Kim et al. 2015	Korea	case-control	NA	laparoscopic/laparotomy population	55/33	Urine	OR	MEHHP, MEOHP, MnBP, MBzP, MECPP	diagnosed using laparoscopy
Upson et al. 2013	USA	case-control	18–49	general population	92/195	Urine	OR	MEHP, MEHHP, MEOHP, MECPP, MBzP, MEPMIBP, MnBP	International Classification of Disease 9th Revision (ICD-9) diagnostic codes 617.0–617.5, 617.8–617.9, excluding adenomyosis
Kim et al. 2011	Korea	case-control	NA	laparoscopic/laparotomy population	97/169	Plasma	OR	MEHP, DEHP	identified by preoperative ultrasonography, and the extent of the disease was staged according to the guidelines of the American Society for Reproductive Medicine

Abbreviations: OR, odds ratio; MEP, monoethyl phthalate; MnBP, mono-n-butyl phthalate; MEHP, mono(2-ethylhexyl) phthalate; MEHHP, mono (2-ethyl-5-hydroxyhexyl) phthalate; MEOHP, mono(2-ethyl-5-oxohexyl) phthalate; MMP, monomethyl phthalate; MBzP, monobenzyl phthalate; 5oxo-MEHP, mono-(2-ethyl-5-oxo-hexyl) phthalate; 5OH-MEHP, mono-(2-ethyl-5-hydroxyhexyl) phthalate; MBP, monobutyl phthalate; MIBP, mono (2-isobutyl phthalate); MECPP, mono-(2-ethyl-5-carboxypentyl) phthalate; MOP, monooctyl phthalate; MNP, monoisonoyl phthalate; DEHP, di (2-ethylhexyl) phthalate; NA, not available.

**Table 2:** The Newcastle-Ottawa quality assessment of included studies.

Study	Year	Study Design	Selection	Comparability	Outcome/Exposure	Score *
Itoh et al.	2009	Case-control	★★★	★	★★	6
Huang et al.	2010	Case-control	★★	★	★★★	6
Weuve et al.	2010	Cross-sectional	★★★	★★	★★	7
Buck Louis et al. (S)	2013	Cohort	★★★★	★	★★	7
Buck Louis et al. (G)	2013	Cohort	★★★★	★	★★	7
Kim et al.	2015	Case-control	★★	★★	★★★	7
Upson et al.	2013	Case-control	★★★	★	★★★	7
Kim et al.	2011	Case-control	★★	★	★★★	6

* For cohort and case-control study, the score ranged from 0 to 9 (selection ≤ 4, comparability ≤ 2, outcome or exposure ≤ 3); for cross-sectional study, the score ranged from 0 to 10 (selection ≤ 5, comparability ≤ 2, outcome ≤ 3).

**Table 3:** Results of the Meta-Analyses of Studies of the Association between mono- (2-ethyl-5-hydroxyhexyl) phthalate (MEHHP) and Endometriosis.

Group and Subgroup	No. of Studies	OR (95% CI)	*p*-Value	I² (%)
All studies	6	1.246 (1.003, 1.549)	0.111	44.1
Study design				
case-control	3	1.508 (0.949, 2.397)	0.381	0
cohort	2	1.472 (0.753, 2.879)	0.028	79.2
cross-sectional	1	1.070 (0.880, 1.210)		
Study population				
laparoscopic/laparotomy population	3	1.337 (0.875, 2.043)	0.184	40.9
general population	3	1.327 (0.831, 2.117)	0.064	63.5
Region				
USA	4	1.170 (0.949, 1.442)	0.138	45.6
Asia	2	1.786 (1.005, 3.172)	0.323	0

Abbreviations: OR, odds ratio; CI, confidence interval.

**Table 4:** Results of the meta-analyses of studies of the association between mono(2-ethylhexyl) phthalate (MEHP) and Endometriosis.

Group and Subgroup	No. of Studies	OR (95% CI)	*p*-Value	I² (%)
All studies	7	1.089 (0.858, 1.383)	0.023	59.2
Study design				
case-control	4	1.025 (0.836, 1.258)	0.331	12.3
cohort	2	1.596 (0.770, 3.306)	0.067	70.1
cross-sectional	1	0.440 (0.190, 1.020)		
Study population				
laparoscopic/laparotomy population	4	1.067 (0.954, 1.194)	0.287	20.5
general population	3	0.884 (0.304, 2.569)	0.005	81.2
Region				
USA	4	0.982 (0.530, 1.819)	0.008	74.8
Asia	3	1.020 (1.003, 1.038)	0.451	0

Abbreviations: OR, odds ratio; CI, confidence interval.

**Table 5:** Results of the meta-analyses of studies of the association between monoethyl phthalate (MEP) and endometriosis.

Group and Subgroup	No. of Studies	OR (95% CI)	*p*-Value	I² (%)
All studies	6	1.073 (0.899, 1.282)	0.423	0
Study design				
case-control	3	1.493 (0.802, 2.778)	0.251	27.7
cohort	2	1.015 (0.834, 1.236)	0.868	0
cross-sectional	1	1.120 (0.580, 2.170)		
Study population				
laparoscopic/laparotomy population	3	1.061 (0.778, 1.448)	0.305	15.7
general population	3	1.264 (0.838, 1.907)	0.402	0
Region				
USA	4	1.057 (0.879, 1.271)	0.434	0
Asia	2	1.179 (0.471, 2.951)	0.172	46.3

Abbreviations: OR, odds ratio; CI, confidence interval.

**Table 6:** Results of the Meta-Analyses of Studies of the Association between monobenzyl phthalate (MBzP) and Endometriosis.

Group and Subgroup	No. of Studies	OR (95% CI)	*p*-Value	I² (%)
All studies	7	0.976 (0.810, 1.176)	0.503	0
Study design				
case-control	4	1.116 (0.790, 1.577)	0.650	0
cohort	2	1.018 (0.605, 1.715)	0.120	58.5
cross-sectional	1	1.160 (0.580, 2.330)		
Study population				
laparoscopic/laparotomy population	4	0.896 (0.727, 1.103)	0.650	0
general population	3	1.378 (0.908, 2.091)	0.822	0
Region				
USA	4	1.067 (0.769, 1.483)	0.223	31.6
Asia	3	1.038 (0.711, 1.516)	0.669	0

Abbreviations: OR, odds ratio; CI, confidence interval.

**Table 7:** Results of the Meta-Analyses of Studies of the Association between MEOHP and Endometriosis.

Group and Subgroup	No. of Studies	OR (95% CI)	*p*-Value	I² (%)
All studies	6	1.282 (0.874, 1.881)	0.053	54.1
Study design				
case-control	3	1.419 (0.811, 2.484)	0.273	22.9
cohort	2	1.489 (0.693, 3.198)	0.018	82.2
cross-sectional	1	0.620 (0.270, 1.440)		
Study population				
laparoscopic/laparotomy population	3	1.241 (0.782, 1.970)	0.170	43.6
general population	3	1.252 (0.574, 2.731)	0.040	68.9
Region				
USA	4	1.195 (0.754, 1.896)	0.053	60.9
Asia	2	1.643 (0.620, 4.356)	0.121	58.5

Abbreviations: OR, odds ratio; CI, confidence interval.
